# Penile cancer: about ten cases at the University Hospital of Rabat, review of the literature

**DOI:** 10.11604/pamj.2015.22.53.6563

**Published:** 2015-09-18

**Authors:** Amine Slaoui, Youness Jabbour, Anouar El Ghazoui, Tarik Karmouni, Khalid Elkhader, Abdelatif Koutani, Ahmed Ibn Attaya

**Affiliations:** 1 Urology B, Ibn Sina University Hospital, Rabat, Morocco

**Keywords:** Penile Cancer, total penectomy, Partial penectomy, lymphadenectomy

## Abstract

The aim of our study was to report the status of penile cancer sites in the urology department at the University Hospital of Rabat and evaluate long-term results of surgical treatment of this cancer. Patients and Methods: Between 1989 and 2015, 10 patients were treated for penile cancer. 10 cases were retrospectively reviewed and the following data were recorded: mode of revelation, seat, staging, TNM stage, treatment, evolution and survival. The mean age of patients was 58,1 years (48-81 years). All patients had squamous cell carcinoma of the penis. Six patients had a partial amputation of the penis, and three patients underwent total amputation. The median size of the lesion was 4.25 cm (1.5-8 cm). All tumors had a distal seat (gland- Furrow balanopreputial), 8 were localized and non-invasive (PT1 - PT2) and 2 had infiltrated the urethra (PT3). Four patients had lymph node localization. A single bilateral lymphadenectomy was performed and was positive only on one side, with a node <3 cm and no extracapsular extension. Two patients were referred for chemotherapy, a neoadjuvant referred to basic (Bleomycin - Methotrexate, Cisplatin) the other in a palliative goal. Median follow-up was 42 months (6 -72mois). Four patients died, one of which was presented immediately with metastatic mode. Six patients were alive at last node or local recurrence negative. Cancer of the penis seems rare in Morocco. His oncologic and functional outcomes (sexual and urinary) depend on the precocity of the treatment. The surgery of lymph node resection with lymphadenectomy remains the reference treatment.

## Introduction

Tumors of the penis are the rarest tumors of the genitourinary system, it represents 0.5% of malignant tumors of man [1]. Penile carcinoma is mostly a squamous cell carcinoma (SCC) but other types of carcinoma exist as well [2]. The incidence of penile cancer increases with age [3], with an age peak during the sixth decade of life. However, the disease does occur in younger men.

Several risk factors for penile cancer have been identified by a review of the literature published since 1966.One of the most important risk factor for developing penile canceris the Human papilloma virus infection. It seems that neonatal circumcision reduces the incidence of penile cancer in countries and cultures where this is routinely practiced. Squamous cell carcinoma can have several clinical aspects: ulcerating or budding, localized or invading the whole structure of the penis.

To appreciate the evolution and expansion of these tumors, several diagnostic tests have been proposed: Ultrasound, CT or MRI and more recently FDG-positron emission tomography. There are a variety of treatment modalities for the penile cancer and are always adapted to TNM stage, tumor grade. The prognosis is pejorative: 80% 5-year survival for N0 and N + 50%.

## Methods

This is a retrospective study interesting ten patients. The following data were recorded: history, diagnosis, mode of revelation, the seat height, balance sheet preparation extension stage (TNM 2009), rank, the margin of resection and therapeutic attitude tick. The patients were seen every 3 months during the first year and then every 6 mounth in the absence of oncological recurrence.

## Results

**Figure 1:**
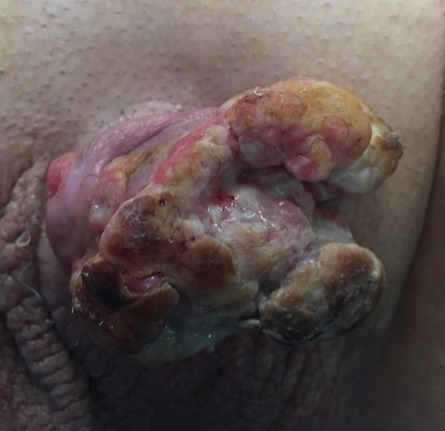
Cauliflower appearance of the tumor

**Figure 2:**
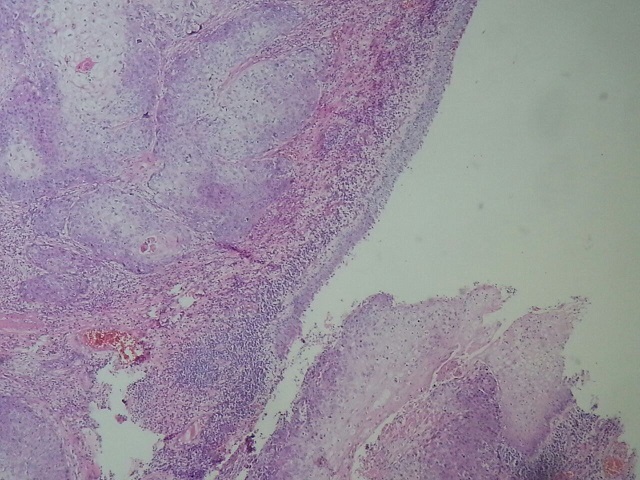
Infiltration of the urethra (HEx100)

**Figure 3:**
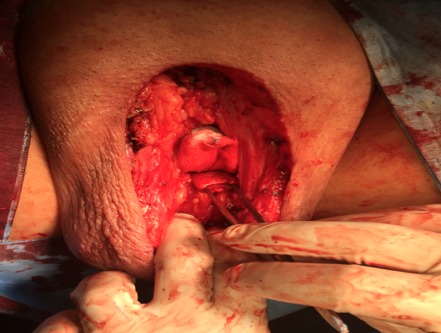
Appearance after total penectomy

**Figure 4:**
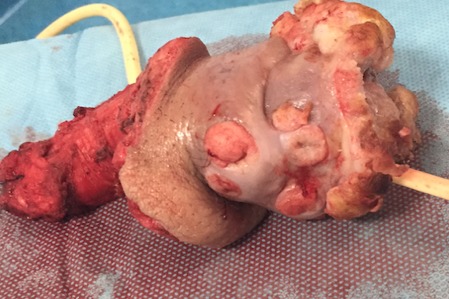
Resected specimen

**Figure 5:**
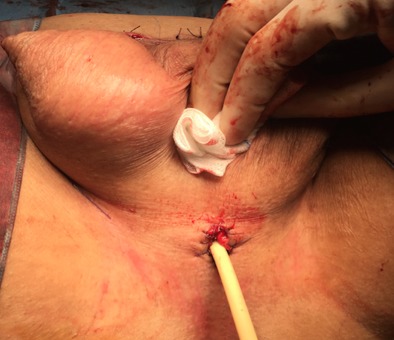
Perineal urethrostomy

Patient characteristics were detailed in Table 1. The mean age at diagnosis was 58,1 years (46-81 years). The mode of revelation in all patients was the presence of a macroscopic lesion (Figure 1), the median tumor size was 5,1cm (1.5-8 cm). All tumors had a distal seat (gland- Furrow balano- preputial), 8 were located and non-invasive (pT1 - pT2) and 2 infiltrated the urethra (pT3) (Figure 2). Three patients had lymph node localization. One of the patients had T3 emblem of inguinal lymph node metastases and liver metastases. Tumor grade was always? 2. Nine patients underwent surgical treatment for early amputation of the penis or partial penectomy (Figure 3, Figure 4, Figure 5). Two patients were referred for chemotherapy, a neo referred based adjuvants (Bleomycin - Methotrexate, Cisplatin) the other in a palliative goal. Median follow-up was 46 months (6 -72mois) 4 patients died Table 1.

**Table 1:** Main characteristics of patients

	P1	P2	P3	P4	P5	P6	P7	P8	P9	P10
Age Diagnosis	55 years	81 years	58 years	48 years	49 years	72 years	46 years	59 years	55 years	58 years
location	glans	Balanopreputial groove	balanopreputial groove	glans	glans	body	glans	glans	glans	glans
size	1,5 cm	5 cm	3 cm	4 cm	5 cm	8 cm	3 cm	3 cm	4 cm	6 cm
PT	PT1	PT2	pT2	PT1	PT1	PT3	PT1	PT1	PT1	PT3
N	N0	N2	N1	N0	N0	N3	N0	N0	N1	N0
M	M0	M0	M0	M0	M0	M +	M0	M0	M0	M0
G	1	2	1	1	1	-	1	1	1	2
Emboli V/L	V0	-	V0/LO	-	-	-	-	-	-	-
Preoperativeassessment	CT	CT	MRI	CT	CT	CT	CT	CT	CT	CT
Precancerous lesion or HPV infection	+	-	-	-	-	-	-	-	-	+
Resectionmargin	-	-	-	-	-	-	-	-	-	-
treatment	Partial penectomy	Total penecto my + neoadjuvant chemo	Total penectomy	Partial penectomy	Partial penectomy	Palliative chemo	Partial penectomy	Partial penectomy	Partial penectomy	Total penectomy
follow	5 years	1 year	3 years	5 years	6 years	6 mounths	4 years	5 years	2 years	-
recidivism	no	no	No	no	no	no	no	no	no	-
death	yes	yes	Yes	no	no	yes	no	no	no	no

## Discussion

**Figure 6:**
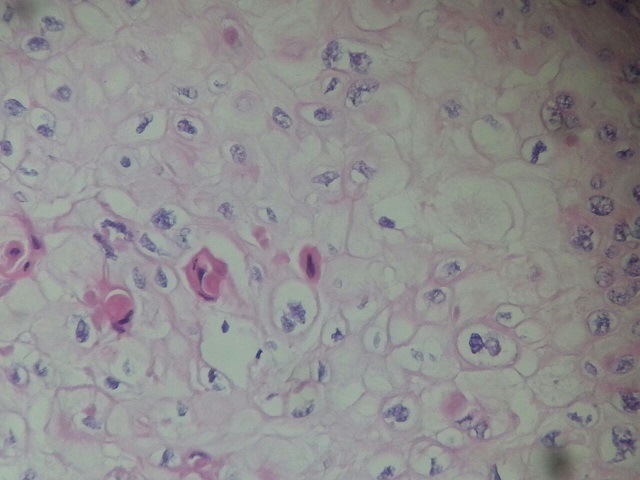
Dyskeratotic cells with tumor proliferation (HEx400)

**Figure 7:**
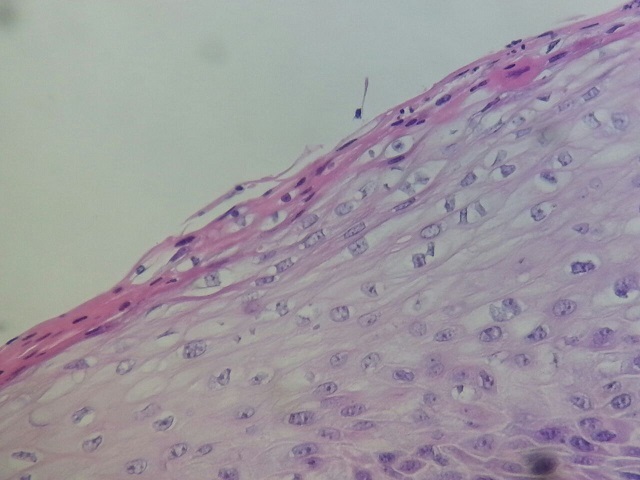
Cutaneous coatings adjacent to the tumor showing parakeratosis. Koilocytespresence (HEx200)

The average age of cancer diagnosis in our study (58,1 years) is consistent with the literature: incidence maximum occurs after 50 years [3]. The location was most often distal (prepuce and glans) according to what is described. One of the most important risk factor for developing penile cancer is the Human papilloma virus infection. It has been identified in 70-100% of intraepithelial neoplasia and in 30-40% of invasive penile cancer tissue samples [4] HPV subtypes most commonly found in penile cancer are types 16 and 18 [5] (Figure 6, Figure 7).

Penile carcinoma is most frequent in uncircumcised males than in those circumcised in infancy, for example: Non-Jewish versus Jewish population in the West and non-Muslim versus Muslim population in India [6]. Circumcision at birth however, does not always protect an individual from developing penile cancer. Also circumcision beyond infancy is also not protective against the development of penile cancer [7]. An other risk factor of the development of invasive penile cancer is the Phimosis [8], probably due to associated chronic infection. A further risk factor suggested by epidemiological studies is cigarette smoking, which is associated with a 4.5-fold increased risk (95%) [9].

Low socio-economic status and a low level of education are other epidemiological factors associated with penile cancer. In our study, only two cases of HPV have been diagnosed on biopsy by the presence of malpigiennes cells infected with the virus who take an aspect quite pathognomonic called koilocyte (large cells with vacuolated cytoplasm with enlarged nuclei and hyperplastic sometimes multinucleated).

Squamous cell carcinoma accounts for more than 95% of cases of malignant diseases of the penis. Other malignant lesions of the penis unrelated to penile SCC are melanocytic lesions, mesenchymaltumours, lymphomas and secondary tumours, i.e. metastases. These are all much less common than penile SCC. Aggressive sarcoma of different types occurring in the penis have been reported.

In our study assessing the local expansion of the penis was based on the penis examination and ultrasound. Ultrasound can give information about infiltration of the corpora [10]. Magnetic resonance imaging (MRI) in combination with an artificial erection with prostaglandin E 1 can also be used for excluding tumour invasion of the corpora cavernosa if organ-preservation is planned and preoperative decisions are needed [11].

All patients had a deep biopsy for histological confirmation and appreciation of the depth of infiltration. The areas of palpation for lymph node assessmentshould be systematic and bilateral. If lymphadenopathy palpated, we should do a needle aspiration cytology of (the) node (s), associated with ultrasound guidance. She increased the micrometastases detection rates in the sentinel lymph node biopsy but few expert center used in practice. When the clinical examination is difficult (obese patients) or if clinical inguinal lymph node involvement (evaluation of pelvic areas) Inguinal and pelvic CT scan isrecommended. It is important to notice that there is no pelvic lymph node involvement that has been demonstrated without inguinal lymph node involvement [12].

The imagery is not essential for normal inguinal lymphadenopathy area without palpated. All patients had at least one abdominopelvic CT scan to define the nodal status in the most comprehensive way possible, but one for which an MRI was performed. MRI has proven superior to CT in the detection of inguinal lymph nodes. The sentinel node was most effective in patients CN0 that cN +. It is not recommended in patients with a palpable lymphadenopathy. The nodal risk is material and requires care in case of penile tumor? pT1bG2 or when palpated adenopathy [13].

An assessment of distant metastases should be performed in patients with positive inguinal nodes. There is no established tumour marker for penile cancer. Regarding surgical treatment, six patients with pT1 N0 M0 tumor site in the glans had a partial penectomy, the other three pT2 N2 M0, pT2 N1 M0, pT3 N0 M0 have experienced a complete amputation with negative margins. The last one had a palliative chemo. So following the recommendations of CCAFU, patients with infiltrating lesions? PT2 had surgery by partial or total penectomy. In case of partial amputation, the length of the remaining penis must be at least 3cm [14].

In clinically lymph-node positive patients, surgical staging by inguinal lymphadenectomy is indicated. Radical inguinal lymphadenectomy carries a significant morbidity related to problems of lymph drainage from the legs and wound healing. While morbidity can be as high as 50% [15]. Wound infections (1.2-1.4%), skin necrosis (0.6-4.7%) lymphedema (5-13.9%) and lymphocele formation (2.1-4%) were the most commonly reported complications in recent series. Therapeutic radical inguinal lymphadenectomy can be life-saving but it may be underused for fear of associated morbidity [16]. The superficial inguinal lymphadenectomy modified involves ablation of superficial inguinal nodes located medial to the great saphenous vein [17]. Morbidity is low at about 6.8% of early complications and 3.4% of late complications [18]. If a modified inguinal lymphadenectomy is performed and confirmed lymph node involvement (or extemporaneous final review), it should always be taken for totalization (lymphadenectomy).

In our study, we performed a single bilateral inguinal lymph node dissection, the dissection was negative right and left we found a single node metastases <3 cm without capsular which did not require a pelvic lymphadenectomy ipsilateral metastasis is recommended if there are more than two nodes or metastasized to a lymph node capsular.

Cancer of the penis is a little chemo-sensitive tumor chemotherapy and no product has the marketing authorization (MA) for use in cancer of the penis. Overall, the results of neoadjuvant or adjuvant chemotherapy does not suggest the interest on survival and local control, given the small number of cases in the series and the absence of randomization.

Two of our patients were referred for chemotherapy, one (pT2N2M0) to neoadjuvant referred to basic (Bleomycin - Methotrexate, Cisplatin) in the general condition does not allow to make a inguinal lymphadenectomy, the other (pT3N3M +) in a palliative goal. There is no consensus in the manner and frequency of monitoring. The follow-up interval and strategies for patients with penile cancer are directed by the initial treatment of the primary lesion and regional lymph nodes. In a multicentre study, during the first two years 74.3% of all recurrences, 66.4% of local recurrences, 86.1% of regional recurrences and 100% of distant recurrences were detected. In the same study, 92.2% of all recurrences occurred within the first 5 years and all recurrences seen after 5 years were local recurrences or new primary lesions. Therefore, an intensive follow-up regimen during the first 2 years is rational, with less intensive follow- up needed thereafter for a minimum of 5 years. Generally, follow-up should continue thereafter but may be omitted in well-educated and motivated patients who reliably continue to carry out regular self-examination [19].

Our patients had clinical monitoring penile and lymph nodes, quarterly in the first year and then every six months in the absence of oncological recurrence.

## Conclusion

The low incidence of penile tumors in the general population remains a real obstacle to the publication of consistent series of patients likely to generate well codified therapeutic management. Human papilloma virus infection is an important risk factor for developing penile cancer. Neonatal circumcision reduces the incidence of penile cancer. The treatment of cancers of the penis is usually surgical roughly in combination with chemotherapy in the case of lymph node. The main prognostic factor is lymph node involvement justifying appropriate care from diagnosis.
